# A New Threat Emerges: The First Detection of GVIII Genotype IBV in China and the Urgent Need for Molecular Surveillance

**DOI:** 10.1155/tbed/8699584

**Published:** 2026-09-08

**Authors:** Jiao Cai, Hongze Pang, Rui Xu, Chenchen Meng, Shiyan Xu, May Myat Thu, Yifan Zhang, Mengjiao Guo, Chengcheng Zhang, Zongyi Bo, Shouchang Zhou, Yantao Wu, Xiaorong Zhang

**Affiliations:** ^1^ Jiangsu Co-Innovation Center for the Prevention and Control of Animal Infectious Disease and Zoonoses, College of Veterinary Medicine, Yangzhou University, Yangzhou, Jiangsu, 225009, China, yzu.edu.cn; ^2^ Huayu Agricultural Science And Technology Co. Ltd, Handan, Hebei, 056000, China

**Keywords:** complete genome, GVIII, IBV, pathogenicity, recombination

## Abstract

In 2025, an infectious bronchitis virus (IBV) strain of the GVIII genotype was identified and isolated from a poultry farm in Hebei Province, China, representing the first documented detection of this IBV genotype within the country to date. Nucleotide homology analysis revealed that the S1 gene of this isolate shares 62.2%–96.9% sequence identity with previously reported GVIII‐type strains from other countries. Comprehensive phylogenetic analysis of the complete genome indicated that this strain originated from a single recombination event: Its S gene may have been derived from the GVIII‐1 lineage strain IBV/Ck/USA/CA/21‐1883, while the remaining genomic regions were likely acquired from the GI‐19 lineage strain SD. Pathogenicity evaluation demonstrated that infected flocks exhibited reduced body weight gain, with 40% of chickens showing signs of diarrhea. Necropsy findings included hemorrhagic lesions at the proventriculus‐gizzard junction and within the bursa of Fabricius. Moreover, 85% of infected hens developed severe oviduct hypoplasia, and 90% showed a reduction in follicular number. The isolate described in this study constitutes the first detection of a GVIII genotype IBV strain in China, although its route of introduction remains undetermined. The emergence of this strain highlights the necessity for sustained molecular surveillance of IBV and the development of genotype‐matched vaccines.

## 1. Introduction

Infectious bronchitis virus (IBV) constitutes a highly significant and economically detrimental pathogen in poultry [1, 2]. This pathogen not only induces respiratory disease but also contributes to kidney injury, gastrointestinal dysfunction, “false layer” syndrome in laying flocks, decreased egg production, and deteriorated egg quality, causing sustained economic losses to the global poultry industry [3]. IBV is classified within the genus *Gammacoronavirus* and is a single‐stranded positive‐sense RNA virus, featuring a genome ~27.6 kb in length [4–6]. Its characteristically high mutation rate and capacity for homologous recombination drive frequent genetic evolution and antigenic variation, leading to the continuous emergence of novel genotypes [7, 8]. This phenomenon presents substantial challenges for IBV prevention, control, and vaccine development [9–12].

Genotyping of IBV is primarily performed based on the S1 subunit of the viral surface spike glycoprotein gene [13, 14]. According to the taxonomic classification system established by Valastro et al., global IBV strains are categorized into 10 genotypes (GI to GX) and more than 40 distinct lineages [15, 16]. In China, the epidemiological landscape of IBV is notably intricate. Substantial evidence from multiple studies indicates that the QX type (GI‐19 lineage) has become the predominant type since the late 1990s, while the mass type (GI‐1 lineage), 793/B type (GI‐13 lineage), TW‐I type (GI‐7 lineage), and TC07‐2 type (GVI‐1 lineage) also account for a large proportion of currently circulating strains [17–19].

Globally, documented reports of the GVIII genotype IBV remain relatively scarce. The first GVIII genotype strain, PA/1220/98, was isolated in the United States and subsequently triggered a widespread outbreak in Canada [20]. Following this, the strain IBV/Ck/USA/CA/21‐1883 was identified in California, USA, and has been classified, together with PA/1220/98, within the GVIII‐1 lineage [21]. In 2016, a strain was isolated from a poultry farm in Germany, which exhibited merely 80% nucleotide sequence homology with the S1 gene of strain PA/1220/98. Petzoldt et al. designated this strain as CK/DE/IB80/2016 (abbreviated as IB80 for short) and assigned it to the GVIII‐2 lineage. IB80 and genetically similar strains have been widely distributed across multiple European countries, including Germany, Poland, Belgium, France, the Netherlands, Lithuania, Belarus, Russia, and Spain. Furthermore, relevant cases have been documented in Jordan and Saudi Arabia in the Middle East. In 2019, another strain belonging to the GVIII‐2 lineage was isolated in the Netherlands and designated D2860 [22–24]. To date, no detection or prevalence of the GVIII genotype of IBV has been reported in China.

In March 2025, an outbreak was reported at a parent stock farm operated by a layer‐breeder company in Hebei Province. The outbreak manifested primarily as decreased egg production and reduced feed intake, accompanied by a slight increase in flock mortality. Necropsy findings included follicular congestion, degeneration, and rupture, with caseous material present in the oviducts of affected birds. An IBV strain, previously unidentified, was isolated from the flock exhibiting reduced egg production. Metagenomic sequencing facilitated the acquisition of the complete genome of this strain. Phylogenetic analysis revealed that the S1 gene of the strain exhibited the highest homology with strain PA/1220/98 from the GVIII‐1 lineage, marking the first identification of this genotype in China. Over the ensuing 5 months, similar infections occurred successively at three additional farms belonging to the same layer chicken company, with egg production declines ranging from 6.57% to 31.49%. Genomic sequencing indicated that the strains infecting these farms were highly genetically related. This study conducted a systematic genetic and evolutionary analysis and pathogenicity assessment of the IBV strain implicated in this outbreak, aiming to provide a scientific basis for evaluating its transmission risk and formulating evidence‐based monitoring and control strategies.

## 2. Materials and Methods

### 2.1. Ethics Statement

All animal experimental procedures were conducted in accordance with the ARRIVE guidelines 2.0 and approved by the Animal Welfare and Ethical Review Committee of Yangzhou University (202507004).

### 2.2. Animals

Specific pathogen free (SPF) embryonated chicken eggs and SPF White Leghorn chickens were purchased from Jiangsu Boehringer Ingelheim Vital Biotechnology Co., Ltd. (Jiangsu, China).

### 2.3. Isolation of IBV

Clinical samples were collected from a layer chicken farm in Hebei Province, China, where a decline in egg production had occurred. Tissues from the trachea, kidneys, cecal tonsils, and oviducts were collected and placed in a phosphate‐buffered saline (PBS) solution (pH 7.2–7.4) containing 10,000 IU/mL of penicillin and 10 mg/mL of streptomycin [25, 26]. After homogenization and centrifugation, the supernatant was inoculated into the allantoic cavity of 9–11‐day‐old SPF chicken embryos for virus isolation [27–29]. Allantoic fluid was collected after 48 h of incubation at 37°C and tested positive for IBV via reverse transcription quantitative polymerase chain reaction (RT‐qPCR) [17]. The IBV‐positive allantoic fluid was stored at −80°C [30]. For titration, the IBV‐positive allantoic was serially diluted by 10‐fold (upto 10^−7^) in PBS (pH 7.2–7.4). Each dilution was inoculated into five SPF embryos, which were then observed for 144 h postinoculation. The 50% embryo infectious dose (EID_50_) of virus was determined with preceding steps and calculated using the Reed–Muench method.

### 2.4. Complete Genome Sequencing

Total RNA was isolated from chicken embryo allantoic fluid utilizing the Ultra‐Pure RNA Kit (CoWin Biosciences, Beijing, China). Reverse transcription was subsequently conducted with random hexamer primers. Following DNase treatment and purification, second‐strand synthesis was performed. Library construction was achieved using the Nextera XT kit (Tanpu Biotechnology, Shanghai, China), and sequencing was carried out on the NovaSeq 6000 platform (Tanpu Biotechnology, Shanghai, China). Bioinformatic analysis of the resultant data adhered to a previously documented workflow [31–33].

Read quality trimming and adapter removal were conducted utilizing Fastp v0.20.0 [34]. Subsequently, contaminant sequences were filtered out using BBmap v38.51. De novo assembly of the next‐generation sequencing data was performed with SPAdes v3.14.1 and SOAPdenovo v2.04 [35, 36].

### 2.5. Phylogenetic Analysis

A total of 52 complete genome sequences and 84 S1 gene sequences of IBV were retrieved from the National Center for Biotechnology Information (NCBI) database (Supporting Information 1 and Supporting Information 2). The reference sequences were aligned with the complete genome and S1 gene sequences of the present strain utilizing the FFT‐NS‐1 algorithm implemented in MAFFT version 7.0 [37]. Phylogenetic analyses were conducted based on the maximum likelihood (ML) method in MEGA X, with 1000 bootstrap replicates to assess nodal support. The resulting phylogenetic tree was annotated via the interactive tree of life (available at https://itol.embl.de/). Furthermore, separate phylogenetic trees were constructed for the ORF1ab, S, E, M, and N genes using the aforementioned analytical pipeline [38]. Nucleotide homology alignment of this strain with six GVIII genotype IBV S1 gene sequences was performed using MegAlign.

### 2.6. Recombination Analysis

Potential recombination events in the complete genome of this strain were detected using the Recombination Detection Program 4 (RDP4, version 4.101). Detection of recombination was performed using seven default methods (RDP, GENECONV, BootScan, Maxchi, Chimaera, SiScan, and 3Seq), with a significance threshold of *p* < 1 × 10^−12^ [39]. A recombination signal was regarded as statistically significant only if it was supported by at least five of these methods [4, 6, 40]. For the visualization of potential recombination breakpoints, a similarity plot was constructed using SimPlot v3.5.1 [41].

### 2.7. Animal Experiments

Eighty 10‐day‐old SPF female chicks were randomly assigned to an infected group (*n* = 40) and a PBS negative control group (*n* = 40). Each chick in the infected group was inoculated via intranasal and ocular routes with 0.1 mL of the isolated strain at a challenge dose of 10^5^ EID_50_ per chick; the control group received an equal volume of PBS administered through the same routes. The physiological status of chicks was observed daily, clinical signs were monitored, and individual observation and evaluation were performed for each chick. Infection was confirmed upon observation of any of the following criteria [42]: (1) depression, anorexia, or huddling; (2) nasal discharge, head shaking, or respiratory symptoms; (3) diarrhea or dehydration; and (4) mortality. On days 3, 6, 9, and 12 postchallenge, five chicks were randomly selected from each group for the collection of oral and cloacal swabs to assess viral shedding. Following euthanasia, necropsy was performed to examine gross lesions, and samples from the trachea, lungs, liver, spleen, kidneys, glandular stomach, muscular stomach, cecal tonsils, and bursa of Fabricius were collected for viral load quantification and histopathological analysis. At 156 days of age, the remaining 20 chickens were euthanized and necropsied to evaluate lesions within the reproductive system and quantify alterations in follicle numbers.

### 2.8. Viral Load Quantification by Real‐Time RT‐PCR

Viral RNA was extracted from tissue homogenates, throat swabs, and cloacal swabs, which were resuspended in 0.5 mL of sterile PBS, collected from both infected and control groups utilizing the aforementioned RNA extraction kit. RT‐qPCR was subsequently conducted, as described previously. For quantification of the IBV viral RNA load, a standard curve was established using a standard plasmid [17].

### 2.9. Histopathological Analyses

On the 6th day postvirus challenge, tissue specimens collected from the trachea, lungs, and kidneys were fixed in a 4% formalin solution, embedded in paraffin, and sectioned into 4‐μm‐thick slides. Following standard protocols, these sections were stained with hematoxylin and eosin (H&E) [43]. Tissue architecture was examined under a light microscope to evaluate pathological lesions, and representative images were captured from regions exhibiting characteristic lesions.

### 2.10. Observation of Tracheal Ciliary Morphology

On the 6th day postvirus challenge, tracheal rings measuring 1–2 mm in length were harvested from the upper (*n* = 3), middle (*n* = 4), and lower (*n* = 3) regions of the trachea. Ciliary beating was examined under a light microscope and assigned scores according to established criteria [44]. The scoring system was defined as follows: ciliary activity in the range of 0%–25% was assigned a score of 4 points; 25%–50% corresponded to 3 points; 50%–75% to 2 points; 75%–99% to 1 point; and 100% activity was scored as 0 points.

A tracheal strip measuring 1 mm in width and 3–4 mm in length was excised and immersed in a 2.5% glutaraldehyde solution, followed by fixation at 4°C for 12 h. After rinsing with PBS, the sample was subjected to sequential ethanol dehydration and drying. Subsequently, gold sputtering was applied for conductive coating, and the ultrastructure of the tracheal mucosal surface was examined using a scanning electron microscope (GeminiSEM 300).

### 2.11. Clinical Monitoring

Following the initial outbreak, ongoing disease surveillance was implemented across the company’s multiple poultry farms. Specimens were obtained from flocks exhibiting clinical symptoms for subsequent virus isolation. The isolated IBV strains underwent nucleotide sequence alignment against the S1 gene sequence of the primary isolate to evaluate genetic relatedness. This approach facilitated a comprehensive assessment of IBV prevalence and transmission dynamics within the company’s farm network.

### 2.12. Statistical Analysis

For all statistical analyses, the threshold for statistical significance was defined as *p* < 0.05. For parametric data, results are expressed as mean ± standard deviation (SD) or as median (range). All statistical analyses were conducted using GraphPad Prism version 9.0 (GraphPad Software, San Diego, CA, USA).

## 3. Results

### 3.1. S1 Genetic Sequence Analysis

**Figure 1:**
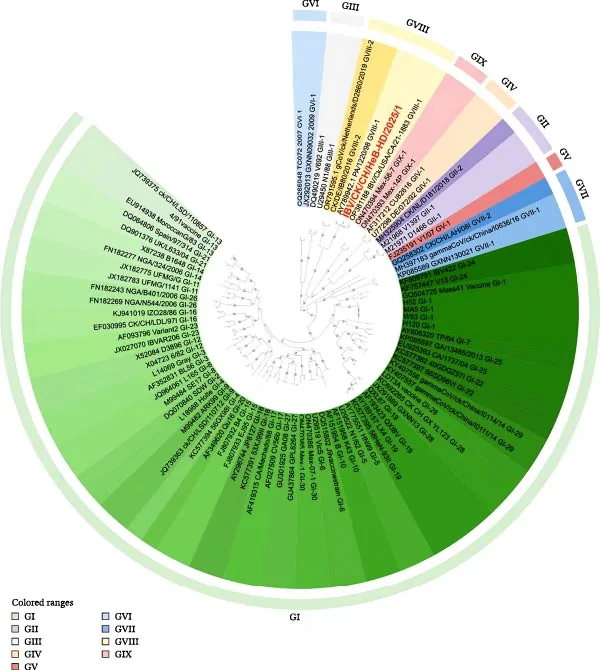
Phylogenetic tree based on the S1 gene sequences of the isolates and the reference strains.

In 2025, an IBV strain was isolated from a poultry farm in Hebei Province, China, and designated as IBV/CK/CH/HeB‐HD/2025/1 (hereinafter referred to as HD251). The complete genome sequence (Accession Number PZ205637) and the S1 gene sequence (Accession Number PZ205638) were deposited in the GenBank database. Phylogenetic analysis of the S1 gene demonstrated that HD251 clusters within a monophyletic clade alongside established GVIII genotype strains, confirming its classification as a member of the GVIII genotype (Figure 1).

After performing an S1 gene nucleotide sequence homology comparison between the isolated strain and four GVIII genotype strains—from the United States, Germany, and the Netherlands—that have been uploaded to GenBank to date (IBV/Ck/USA/CA/21‐1883, PA/1220/98, CK/DE/IB80/2016, and gCoV/ck/Netherlands/D2860/2019), it was found that the nucleotide sequence homology between strain HD251 and the other GVIII strains ranged from 79.9% to 96.9%, while the amino acid sequence homology ranged from 75.7% to 96.2% (Table 1). Among these, the highest homology was observed with PA/1220/98, a GVIII‐1 lineage strain isolated from North America, with 96.9% nucleotide sequence homology and 96.2% amino acid sequence homology. In contrast, nucleotide and amino acid sequence homology with the GVIII‐2 lineage strains CK/DE/IB80/2016 and gCoV/ck/Netherlands/D2860/2019 did not exceed 80%. These findings indicate that strain HD251 belongs to the GVIII genotype, specifically the GVIII‐1 lineage.

**Table 1:** Nucleotide and amino acid sequence alignments of the isolated strain with the S1 gene of existing GVIII genotype IBV.

Virus name	Nucleotide identity (%)				
	IBV/Ck/USA/CA/21‐1883	PA/1220/98	CK/DE/IB80/2016	gCoV/ck/Netherlands/D2860/2019	IBV/CK/CH/HeB‐HD/2025/1
IBV/Ck/USA/CA/21‐1883	—	97.1	80.2	79.6	95.2
PA/1220/98	96.2	—	80.8	80.8	96.9
CK/DE/IB80/2016	76.6	76.8	—	97.5	80.0
gCoV/ck/Netherlands/D2860/2019	75.7	77.1	95.9	—	79.9
IBV/CK/CH/HeB‐HD/2025/1	94.4	96.2	75.4	75.8	—
	Amino acid identity (%)				

### 3.2. Complete Genome Phylogenetic Analysis

**Figure 2:**
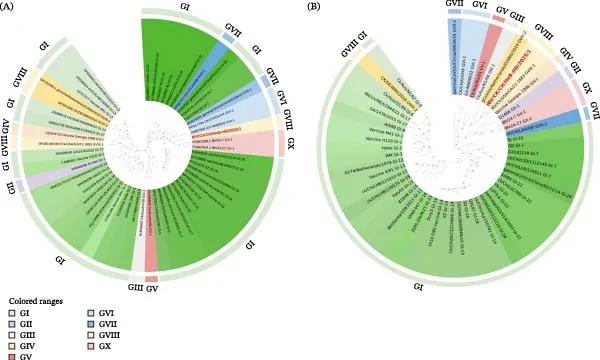
Phylogenetic trees constructed from the complete genome and individual genes of the isolates: (A) phylogenetic tree based on the complete genome sequences and (B) phylogenetic tree derived from the S gene sequences.

To elucidate the evolutionary origin of HD251, a ML phylogenetic tree was constructed based on complete genome sequences. The phylogenetic analysis demonstrated that HD251 does not cluster within any known clade of GVIII genotype strains, implying that this isolate likely represents a novel, domestically circulating strain. Phylogenetic examination of the encoded proteins revealed complex evolutionary relationships among the corresponding genes (Figure 2). Specifically, the ORF1ab and N genes showed close homology to GI‐19 (CK/CH/JS/CZ210840) (Supporting Information 3 and Supporting Information 5). In contrast, the S gene formed a distinct clade with GVIII‐1 (IBV/Ck/USA/CA/21‐1883), indicating that the full‐length spike gene of HD251 is classified within the GVIII group. The M gene was closely related to GI‐19 (ck/CH/LHB/130575) (Supporting Information 4). However, the E gene displayed a relatively close genetic relationship with multiple GI‐22 strains (LDT3‐A, BJ, and partridge/GD/S14/2003), albeit with a bootstrap support of only 34%, suggesting low reliability (Supporting Information 6). These results suggest that HD251 may have undergone recombination or gene transfer events involving different genotypes.

### 3.3. Recombination Analysis

**Figure 3:**
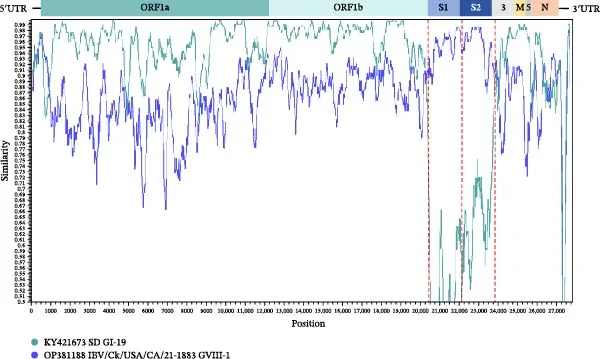
Complete genome recombination analysis of the isolated strain.

To investigate the potential recombination origin of this strain, recombination events within the complete genome were analyzed using RDP4 software. Complete genome recombination analysis indicated that strain HD251 underwent a single recombination event. The major parental strain was identified as the SD strain from the GI‐19 lineage, while the minor parental strain was the IBV/Ck/USA/CA/21‐1883 strain from the GVIII‐1 lineage. Visual analysis employing SimPlot further corroborated these findings, demonstrating that the S gene of strain HD251 originated from the GVIII‐1 lineage strain IBV/Ck/USA/CA/21‐1883, whereas the remaining genomic segments likely derived from the GI‐19 lineage strain SD (Figure 3).

### 3.4. Pathogenicity of the Isolate in Chicks

**Figure 4:**
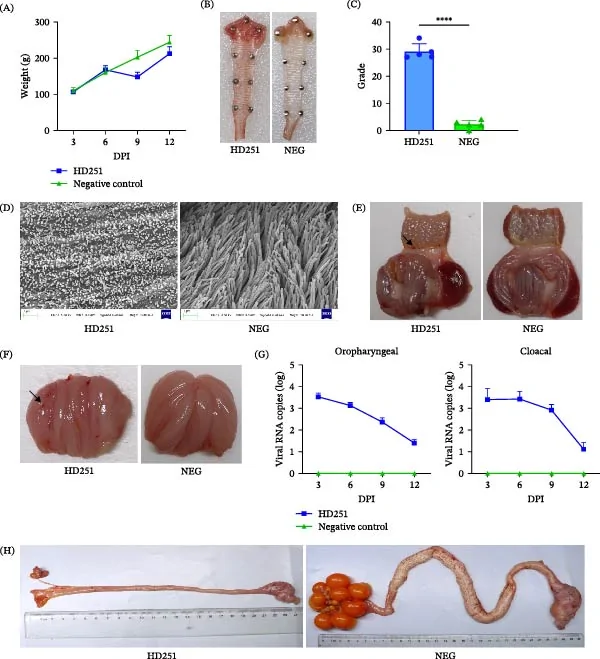
Alterations in body weight, pathological changes in the trachea, glandular stomach, bursa of Fabricius, and oviduct, along with variations in viral excretion following challenge: (A) body weight curve, (B) pathological alterations in the trachea on day 3 postchallenge, (C) tracheal ciliary motility score on day 6 postchallenge, (D) morphological observation of tracheal cilia on day 6 postchallenge, (E) pathological changes in the glandular and muscular gizzards on day 9 postchallenge, (F) pathological changes in the bursa of Fabricius on day 9 postchallenge, (G) dynamics of virus excretion within 12 days postchallenge, and (H) pathological changes in the oviducts of the flock after the onset of egg production. Statistical significance is indicated as follows: 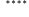 *p* < 0.0001.

**Figure 5:**
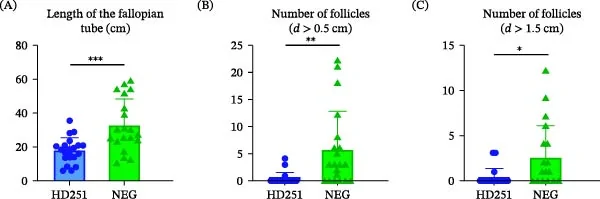
Oviduct length and follicular counts in flocks following the initiation of egg production: (A) variations in oviduct length, (B) alterations in follicle count (diameter > 0.5 cm), and (C) alterations in follicle count (diameter > 1.5 cm). Statistical significance is indicated as follows: 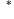 *p* < 0.05, 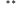 *p* < 0.01, and 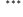 *p* < 0.001.

Ten‐day‐old SPF chickens were infected with HD251 and monitored over a 12‐day period. Clinical signs including lethargy, anorexia, and depression were observed from day 4 postinfection. By day 9 postinfection, a significant reduction in body weight was recorded in the challenged group (Figure 4A), and diarrhea was evident in 40% of the birds; no pronounced respiratory signs were noted. Necropsy revealed marked diffuse hemorrhagic foci in the trachea on day 3 (Figure 4B). By day 6, tracheal mucus secretion was increased, accompanied by severe ciliary damage characterized by extensive inversion, shortening, and loss; the ciliary motility score was significantly elevated (Figure 4C,D). On day 9, hemorrhagic lesions were observed at the glandular‐muscular stomach junction and in the bursa of Fabricius (Figure 4E,F). Pharyngeal viral shedding exhibited a gradual decline in the infected group, whereas cloacal viral shedding peaked on day 6 before gradually decreasing (Figure 4G). Postmortem examination of the oviduct in laying hens revealed severe hypoplasia in 85% of the birds (Figure 4H); the oviduct length was ~14.72 cm shorter compared with the PBS control group (Figure 5A). In 90% of the hens, a significant reduction in follicle number was observed: follicles > 0.5 cm in diameter decreased from an average of 5.95–0.40 (Figure 5B), and follicles > 1.5 cm in diameter decreased from an average of 2.45–0.35 (Figure 5C).

**Figure 6:**
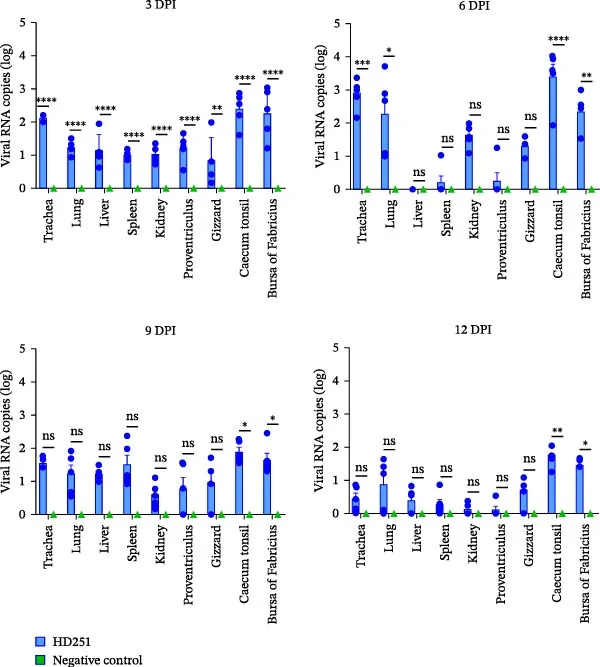
Comparison of viral load between the infection groups and control groups in various organs on days 3, 6, 9, and 12. Statistical significance is indicated as follows: ns = not significant,  ^∗^ = *p* < 0.05,  ^∗∗^ = *p* < 0.01,  ^∗∗∗^ = *p* < 0.001, and  ^∗∗∗∗^ = *p* < 0.0001.

Viral RNA quantification via qRT‐PCR was performed in major tissues and organs of infected chickens—including the trachea, lungs, liver, spleen, kidneys, glandular stomach, muscular stomach, cecal tonsils, and bursa of Fabricius—on days 3, 6, 9, and 12 postinfection (Figure 6). At days 3 and 6 postinfection, viral loads in the trachea and cecal tonsils remained at comparatively elevated levels, consistent with the viral shedding patterns observed in the pharynx and cloaca. Furthermore, in the bursa of Fabricius, viral load was notably high during the early phase of infection (days 3 and 6) and declined by day 9 postinfection. In conjunction with the hemorrhagic lesions observed in the bursa of Fabricius (Figure 4F), this pattern reflects immune‐mediated pathological injury in the host, a phenomenon also documented in infectious bursal disease. Notably, by day 9 postinfection, characteristic hemorrhagic lesions emerged at the junction of the glandular and muscular stomachs, despite low viral detection in this region. This indicates that the pathology at this site may not be principally attributable to direct viral replication but rather may arise from systemic or localized microvascular compromise.

**Figure 7:**
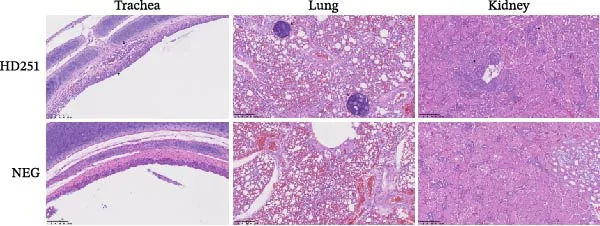
Histopathological changes in the trachea, lungs, and kidneys on the sixth day postinfection.

To elucidate the tissue‐level pathogenic mechanisms of HD251, histopathological examination was conducted on tracheal, pulmonary, and renal specimens harvested at 6 days postinfection (Figure 7). In the infected cohort, the trachea displayed ciliary loss, epithelial desquamation, and submucosal lymphocytic infiltration. The lung tissue presented mild bronchial epithelial disruption and localized inflammatory foci. Renal sections revealed only minor alterations in tubular epithelial cells accompanied by mild inflammatory infiltration. Collectively, these observations demonstrate that HD251 induces merely mild pathological injury in the lungs and kidneys.

### 3.5. Disease Tracking and Surveillance

Over a 1‐year surveillance period conducted across multiple farm sites operated by this poultry enterprise, outbreaks of IBV were identified at a total of four distinct farm locations. Testing and isolation procedures confirmed that all recovered strains pertained to the IBV genotype GVIII, designated as IBV/CK/CH/HeB‐HD/2025/2 (Accession Number PZ263076), IBV/CK/CH/HeB‐HD/2025/3 (Accession Number PZ263077), IBV/CK/CH/HeB‐HD/2025/4 (Accession Number PZ263078), and IBV/CK/CH/HeB‐HD/2025/5 (Accession Number PZ263079). Comparative analysis of nucleotide sequences between HD251 and the S1 gene sequences of the aforementioned isolates revealed a high degree of homology (99.7%–99.9%). In conjunction with an epidemiological assessment of outbreak timing across different farm zones, these findings strongly suggest that the dissemination of IBV genotype GVIII within the farm complex likely resulted from interfarm transmission and subsequent coinfection events.

## 4. Discussion

This study provides preliminary evidence indicating that the GVIII genotype of IBV has been introduced into Chinese poultry populations, thereby extending its previously documented geographical distribution from Europe, the Middle East, and the Americas to Asia. Comprehensive genomic phylogenetic analysis reveals that only the S gene of the HD251 strain belongs to the GVIII lineage, whereas the evolutionary relationships of the other genes are relatively complex. Specifically, the ORF1ab, M, and N genes exhibit closer evolutionary affinities to the GI‐19 lineage, and the E gene shows a closer affinity to the GI‐22 lineage. However, complete genome recombination analysis indicates that strain HD251 underwent only a single recombination event, with the GI‐19 lineage constituting the genomic backbone; its S gene may have originated from the GVIII genotype IBV/Ck/USA/CA/21‐1883, which contradicts the results of the aforementioned phylogenetic analysis. Upon meticulous analysis, it can be inferred that although independent phylogenetic analysis of the E gene reveals its clustering with GI‐22, the support for this branch is low (bootstrap value 34%), and the E gene sequence is relatively short, containing limited phylogenetic signal. Based on comprehensive recombination analysis and the complete genomic architecture, this strain is still classified as a recombinant with a GI‐19 backbone and an inserted S gene of GVIII origin. The placement of the E gene in the phylogenetic tree may be influenced by convergent evolution or the selection of reference strains and does not reflect the true origin of genomic recombination. Xu et al. [45] demonstrated that wild bird migration is a critical transmission route in the dissemination of *Gammacoronaviruses*. Tai et al. [9] investigated the evolutionary relationships and transmission routes of IBV by employing phylogenetic network and recombination analysis on 311 IBV strains, revealing that migratory birds significantly contribute to IBV transmission. Although the exact introduction route remains elusive, given that the GVIII genotype IBV had not been previously detected in China or even in Asia, it is hypothesized that this strain may have been introduced via indirect routes, such as wild bird migration.

Since 2015, this genotype has been identified in multiple countries, including North America, Germany, the Netherlands, Poland, and Spain, and it is capable of infecting both laying hens and broiler chickens. The virus can induce a decline in egg production within affected flocks. In certain regions, infections have also been documented to present with nephritis and respiratory symptoms [21, 22]. Pathogenicity assessment of the HD251 strain demonstrated that infected birds exhibited mild tracheal rales and underdeveloped oviducts, aligning with previously described clinical manifestations associated with the IBV genotype GVIII. In the present study, it was observed that on day 9 postviral challenge, the viral loads in organs including the bursa of Fabricius, glandular stomach, and muscular stomach decreased significantly compared with those detected on days 3 and 6 postchallenge, while histopathological lesions were progressively aggravated. This finding indicates a dissociation between the peak of viral replication and the peak of pathological damage during infection with this strain, suggesting that its pathogenicity may involve a combination of direct viral injury and host immunopathological responses. Integrated with whole‐genome recombination analysis, it was determined that although all genomic regions except the S gene originated from the GI‐19 lineage, the strain did not induce the hallmark nephropathy characteristic of GI‐19 viruses. This supports the inference that the IBV S gene is a critical determinant of tissue tropism and pathogenicity.

Since 2015, the GVIII genotype of IBV has maintained continuous circulation. The PA/1220/98‐like strain of the GVIII‐1 lineage is predominantly prevalent in the United States and Canada, while the IB80‐like strain of the GVIII‐2 lineage is primarily prevalent in Europe; nonetheless, the GVIII genotype has not displaced the currently dominant IBV genotype. Previously reported GVIII strains have generally exhibited low pathogenicity, and their limited tissue tropism may reduce transmissibility or indicate weaker competitive fitness. Notably, although the HD251 strain isolated in this study displays multiorgan tropism, infected flocks frequently show no prominent respiratory or gastrointestinal symptoms during the brooding period, and clinical signs are often subtle. This increases the likelihood of the strain being overlooked in production settings. Nevertheless, its impact on the reproductive system is both insidious and severe, manifesting primarily as pronounced oviduct hypoplasia. Relying solely on clinical observation is therefore insufficient for detecting outbreaks caused by this strain; active surveillance coupled with early serological or molecular testing is essential. Regular monitoring of IBV pathogens and antibody levels should be implemented to inform timely adjustments in immunization protocols and biosecurity measures.

Through a 1‐year longitudinal investigation at an all‐in/all‐out (AIAO) chicken farm, this study elucidated the transmission patterns of IBV genotype GVIII within this production system. While the AIAO model theoretically facilitates the interruption of continuous viral transmission via comprehensive interbatch disinfection, periodic infections were still observed on the farm, suggesting the potential involvement of multiple transmission vectors. Among these, vehicles used for transporting feed and poultry manure may constitute a critical mechanical transmission route, as residual feces or feed remnants can lead to cross‐batch transmission of IBV. Furthermore, the migratory and foraging activities of wild birds may introduce the virus, contaminating the farm environment as well as feed and water sources. Additionally, virus‐laden aerosols disseminated between poultry houses may facilitate short‐ to medium‐range airborne transmission under specific meteorological conditions. The aforementioned propositions regarding potential routes of viral transmission are purely speculative and have not yet been substantiated by definitive empirical evidence. In an AIAO system, it can be inferred that vehicles, wild birds, and airborne transmission collectively contribute to the complex, multipathway transmission network of IBV. Prevention and control measures should include establishing dedicated vehicle decontamination stations and strengthening vehicle decontamination procedures while also installing bird‐proofing facilities and air filtration systems to prevent and interrupt the transmission of IBV.

The detection of IBV genotype GVIII in China underscores the need to reevaluate current diagnostic and surveillance strategies. Most routine IBV detection protocols in China are designed to amplify and distinguish common genotypes. The genetic divergence of IBV genotype GVIII from these established lineages implies that conventional S1‐targeted PCR assays may fail to detect this genotype or yield false‐negative results. Therefore, it is imperative to develop and incorporate genotype‐specific RT‐PCR detection methods for GVIII, which are essential for accurate monitoring of its circulation and prevalence in Chinese poultry populations.

The emergence of the IBV genotype GVIII in China underscores the imperative for proactive strategies in vaccine development and evaluation. Currently, existing vaccines demonstrate substantial genetic divergence from the GVIII strain. Empirical evidence indicates that repeated vaccination on poultry farms where this strain was isolated failed to confer effective immune protection, suggesting significant antigenic disparities between this strain and other IBV genotypes. As the present study did not evaluate vaccine cross‐protection through serological assays, to prevent a widespread outbreak of GVIII genotype IBV, while close monitoring of the transmission and prevalence of this genotype needs to be maintained, systematic evaluation of the cross‐protection against this strain conferred by existing vaccines in combined immunization regimens should be conducted, and a GVIII genotype–specific vaccine should be developed if necessary.

In conclusion, the identification of IBV/CK/CH/HeB‐HD/2025/1 in China expands the known geographical distribution of this genotype and contributes to the complexity of IBV epidemics in China. The distinctive biological characteristics of this strain differentiate it from circulating strains in China. Although this strain was initially isolated from a single poultry farming company, we detected IBV type GVIII at multiple farms in different locations, and the pathological changes were consistent. This supports, to some extent, the generalizability of the findings; however, broader surveillance is still necessary. This research further emphasizes that long‐term molecular surveillance, in conjunction with continued investigations into biological characteristics and vaccine efficacy, is essential for mitigating the risks posed by IBV genotype GVIII and other genotypes that may emerge in the future.

## Author Contributions

Jiao Cai and Hongze Pang conceived, designed, and performed the experiments and analyzed the data. Rui Xu, Chenchen Meng, Shiyan Xu, May Myat Thu, and Yifan Zhang contributed to the experiments. Mengjiao Guo, Zongyi Bo, and Chengcheng Zhang contributed to the data analysis. Jiao Cai drafted the original paper. Xiaorong Zhang helped revise the manuscript. Yantao Wu, Xiaorong Zhang, and Shouchang Zhou supervised the study.

## Funding

This work was supported by the Earmarked Fund for Modern Agroindustry Technology Research System (Grant CARS‐39), the 111 Project (Grant D18007), and the Fund of the Priority Academic Program Development of Jiangsu Higher Education Institutions (PAPD).

## Disclosure

All the authors have read and approved the final manuscript.

## Conflicts of Interest

The authors declare no conflicts of interest.

## Supporting Information

Additional supporting information can be found online in the Supporting Information section.

## Supporting information


**Supporting Information 1** Complete Genome Phylogenetic Tree Database.

**Supporting Information 2** S1 Gene Phylogenetic Tree Database.

**Supporting Information 3** Phylogenetic tree inferred from the ORF1ab gene sequences.

**Supporting Information 4** Phylogenetic tree constructed using the M gene sequences.

**Supporting Information 5** Phylogenetic tree generated from the N gene sequences.

**Supporting Information 6** Phylogenetic tree based on the E gene sequences.

## Data Availability

Research data are not shared.
